# Normal mode analysis in multi-coupled non-Hermitian optical nanocavities

**DOI:** 10.1038/s41598-023-44809-w

**Published:** 2023-10-16

**Authors:** Kyong-Tae Park, Kyoung-Ho Kim, Byung-Ju Min, You-Shin No

**Affiliations:** 1 Department of Physics, Konkuk University, Seoul, 05029 Republic of Korea; 2 Department of Physics, Chungbuk National University, Cheongju, 28644 Republic of Korea

**Keywords:** Engineering, Optics and photonics

## Abstract

Coupled optical cavities are an attractive on-chip optical platform for realizing quantum mechanical concepts in electrodynamics and further developing non-Hermitian photonics. In such systems, an intercavity interaction is often considered as a key parameter to understand the system’s behaviors but its estimation/calculation is typically limited for some simplified systems owing to extended complexities. For example, multi-coupled photonic crystal (PhC) nanocavities exhibiting strong resonances with a large free spectral range can serve as an excellent test-bed to study non-Hermitian optical properties when spatially non-uniform gain is introduced. However, the detailed quantitative analysis such as spectral tracing of cavity normal modes is often limited in commercially available numerical tools because of the required massive computation resources. Herein, we report on a concept of spatial overlap integrals (SOIs) between the eigenmodes in non-coupled PhC nanocavities and utilize them to obtain the intercavity interactions in passively coupled PhC nanocavity systems. With the help of coupling strength factors calculated from SOIs, we were able to fully exploit the coupled mode theory (CMT) and readily trace the detailed spectral behaviors of normal modes in various multi-coupled PhC nanocavities. Full-wave numerical simulation results verified the proposed method, revealing that the characteristics of original eigenmodes from non-coupled PhC nanocavities can act as key building blocks for analyzing the normal modes of multi-coupled PhC nanocavities. We further applied this SOI method to various multi-coupled PhC nanocavities with non-symmetric optical gain/loss distributions and successfully observed the unusual spectral evolution of normal modes and the correspondingly occurring unique non-Hermitian behaviors.

## Introduction

Over the past decade, various coupled optical systems, which are typically composed of a pair of key optical components, have drawn significant attention because they provide an on-chip optical platform for realizing the quantum mechanical concept of parity-time (PT) symmetry^1–19^. Accordingly, unusual optical phenomena, including the symmetry-broken propagation of light and the modal bifurcation/degeneration near exceptional points (EPs), have been successfully observed in various types of coupled optical waveguides^1–8^ and micro- and/or nanocavities^9–19^. It has also shown that the smart control of key parameters allows for in-depth explorations, revealing the emergence of nonlocality effects and complex supermodes, and the formation of spatial and temporal solitons^2,20–24^. In particular, for coupled optical cavities, these counter-intuitive and non-Hermitian behaviors have been demonstrated in systematically controlled manners by introducing an asymmetric optical gain/loss distribution or by leveraging a fine interplay between the gain/loss and the variable intercavity interaction (or coupling)^12–19^. In such systems, the coupled mode theory (CMT)-based master equation, which describes the spectral behaviors and the corresponding phases of systems, dictates the critical importance of intercavity interaction. For example, the relative strength of interaction for a given gain/loss in coupled microcavities determines whether the system’s state is broken or unbroken PT symmetric, exhibiting the ability to control light transmission between two microstructures^13–15^.

In this regard, an in-depth understanding of interstructural interactions in coupled optical cavities has become essential in understanding the overall phenomena and utilizing them for useful optical applications. However, despite successful demonstrations in previous reports, further exploration of the potential of non-Hermitian properties in more extended and complex coupled optical systems, which typically consist of more than one pair of key components, is limited^19^. This is partly because calculating/estimating the intercavity couplings between the components is difficult without constraining them to be symmetrically identical in shape or considering certain interactions to be negligible, which eventually makes it challenging to analyze the behaviors of entire systems from the master equation. Consequently, various design proposals with theoretical studies and experimental demonstrations as well as their unique device applications have not been extensively investigated.

In this paper, we report a concept of the spatial overlap integrals (SOIs) to numerically calculate the interstructural interactions between two optical nanocavities, which can be ubiquitously applicable to more complex coupled nanocavity systems. In particular, we employed two-coupled (or doubly coupled) passive photonic crystal (PhC) nanocavities and quantitatively compared the intercavity couplings obtained from the conventional method and the SOI technique for validation. Furthermore, we applied the technique to more complex non-Hermitian nanocavities with non-symmetric optical gain/loss distributions and investigated their unique spectral and modal behaviors.

## Results

### Intercavity interaction in a coupled cavity system

**Figure 1:**
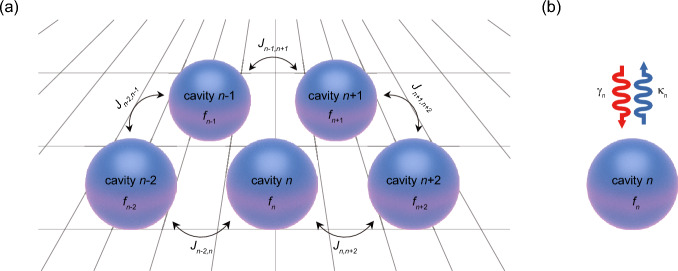
(**a**) Schematic of a coupled cavity system consisting of several element cavities. The individual element cavities are coupled with one another through coupling constants ${J}_{n,m}$, where $n$ and $m$ represent different cavity numbers. (**b**) The *n*th element cavity retains the intrinsic optical gain, ${\gamma }_{n}$, and loss, ${\kappa }_{n}$, respectively.

We begin by considering a coupled cavity system consisting of several element cavities with different intrinsic optical properties (Fig. 1a). Each element cavity supports original eigenmodes when there are no intercavity interactions. An original eigenfrequency of a specific eigenmode of *n*th element cavity can then be written by $f_{n} + i(\gamma_{n} - \kappa_{n} )$ with the time harmonics of $\exp ( - i\omega_{n} t)$ (Fig. 1b). Here, $f_{n}$ is the eigenfrequency determining the spectral position in a dispersion diagram (i.e., $\omega_{n} = 2\pi f_{n}$), and $\gamma_{n}$ and $\kappa_{n}$ are the intrinsic optical gain and loss, respectively. If these individual element cavities are combined into a system to form a coupled cavity, the eigenfrequencies of the system ($f_{{{\text{sys}}}}$) are no longer the same as those of the element cavities. Instead, they are determined by the intercavity interactions parameterized by the coupling constants $J_{n,m}$.

To understand how the interactions determine $f_{{{\text{sys}}}}$, we utilize the concept of coupled mode theory (CMT) and describe the system as the following characteristic matrix equation consisting of several coupled linear differential equations:

1 $$ \frac{i}{2\pi }\frac{d}{dt}\left[ {\begin{array}{*{20}c} \vdots \\ {\begin{array}{*{20}c} {a_{n - 1} } \\ {a_{n} } \\ {a_{n + 1} } \\ \end{array} } \\ \vdots \\ \end{array} } \right] = \left[ {\begin{array}{*{20}c} {} & {} & \vdots & {} \\ {} & {f_{n - 1} + i\left( {\gamma_{n - 1} - \kappa_{n - 1} } \right)} & {J_{n - 1,n} } & {J_{n - 1,n + 1} } \\ \cdots & {J_{n,n - 1} } & {f_{n} + i\left( {\gamma_{n} - \kappa_{n} } \right)} & {J_{n,n + 1} } \\ {} & {J_{n + 1,n - 1} } & {J_{n + 1,n} } & {f_{n + 1} + i\left( {\gamma_{n + 1} - \kappa_{n + 1} } \right)} \\ {} & {} & \vdots & {} \\ \end{array} \begin{array}{*{20}c} {} \\ {} \\ \cdots \\ {} \\ {} \\ \end{array} } \right]\left[ {\begin{array}{*{20}c} \vdots \\ {\begin{array}{*{20}c} {a_{n - 1} } \\ {a_{n} } \\ {a_{n + 1} } \\ \end{array} } \\ \vdots \\ \end{array} } \right] $$

Here, $a_{n}$ represents the amplitude of the eigenmode supported by the *n*th element cavity. The solutions then represent the $f_{{{\text{sys}}}}$, which are expressed by a combination of $f_{n}$, $\gamma_{n}$, $\kappa_{n} ,$ and $J_{n, m}$ (where *n* and *m* = 1, 2, 3, …), and reveal how the intercavity interactions play a critical role on the entire system’s spectral behaviors. Although the current form of the coupled equations exhibits some complexity, several assumptions can be made to simplify it without loss of generality. For example, we first consider identical element cavities with the same eigenfrequency of $f_{0}$. Second, we assign a lossless passive condition to every element cavity ($\gamma_{n} = \kappa_{n} = 0$, for all *n*). Finally, if we apply time-reversal symmetry to the intercavity interactions, Eq. (1) becomes a simply reduced and intuitive form as follows:

2 $$ \frac{i}{2\pi }\frac{d}{dt}\left[ {\begin{array}{*{20}c} {\begin{array}{*{20}c} \vdots \\ {a_{n - 1} } \\ \end{array} } \\ {a_{n} } \\ {\begin{array}{*{20}c} {a_{n + 1} } \\ \vdots \\ \end{array} } \\ \end{array} } \right] = \left[ {\begin{array}{*{20}c} \vdots \\ { \cdots \begin{array}{*{20}c} {f_{0} } & {J_{n - 1,n} } & {J_{n - 1,n + 1} } \\ {J_{n - 1,n} } & {f_{0} } & {J_{n,n + 1} } \\ {J_{n - 1,n + 1} } & {J_{n,n + 1} } & {f_{0} } \\ \end{array} \cdots } \\ \vdots \\ \end{array} } \right]\left[ {\begin{array}{*{20}c} {\begin{array}{*{20}c} \vdots \\ {a_{n - 1} } \\ \end{array} } \\ {a_{n} } \\ {\begin{array}{*{20}c} {a_{n + 1} } \\ \vdots \\ \end{array} } \\ \end{array} } \right] $$

Provided that the necessary information (e.g., number of cavities *n*, eigenfrequency of element cavity $f_{0}$) is given, one can readily solve this equation and obtain $f_{{{\text{sys}}}}$ in the form of $f_{0}$ and $J_{n,m}$. For example, for the simplest case of *n* = 2, the system eigenfrequencies of even ($f_{ + }$) and odd normal modes ($f_{ - }$) are $f_{ \pm } = f_{0} \pm J$. Accordingly, we can analytically understand how the original eigenfrequency is split into two eigenfrequencies of normal modes of the system. In recent years, interesting studies of doubly coupled non-Hermitian cavity systems (i.e., $\gamma_{i} ,\kappa_{i} \ne \gamma_{j} ,\kappa_{j}$) have advantageously utilized the fine interplay between the intercavity coupling and the gain/loss asymmetry. In these studies, the normal mode eigenfrequencies were expressed as $f_{ \pm } = f_{0} \pm (J^{2} - \Delta \gamma^{2} )^{1/2} + i(\gamma_{{{\text{avg}}}} - \kappa )$, where $\Delta \gamma = (\gamma_{2} - \gamma_{1} )/2$ and $\gamma_{{{\text{avg}}}} = (\gamma_{2} + \gamma_{1} )/2$^17^. By independently controlling either the coupling strength or the gain/loss profile, these studies successfully demonstrated unprecedented control over the PT-symmetric phases and their transitions at EPs and observed the correspondingly occurring modal coalescence. Notably, these studies serve as excellent examples of how critical it is to determine the exact values of the coupling constant to understand the characteristic phenomena and their underlying physics. In the next section, we briefly discuss methods for obtaining $J_{n,m}$ in coupled systems.

### Spatial overlap integral method

In many cases, particularly for the aforementioned doubly coupled systems with identical element cavities, a direct method called the frequency method has been widely used to estimate $J$. In this method, one can independently calculate the single-element cavity and the entire system and find the corresponding eigenfrequencies of $f_{0}$ and $f_{{{\text{sys}}}}$. The coupling constant $J$ can then be directly obtained from the solution of the characteristic equation (e.g., for a lossless passive system, $J = (f_{ + } - f_{ - } )/2$). However, despite the successful analysis in those systems, it is not always trivial to obtain $J_{n,m}$ for the systems with *n* ≥ 3. It is in fact impossible to analytically express $J_{n,m}$ in terms of $f_{0}$ and $f_{{{\text{sys}}}}$. Consequently, it becomes more challenging to quantitatively analyze the exact contribution of interaction between individual entities to the overall system’s spectral behaviors.

In this regard, we have focused on an alternative numerical technique called the spatial overlap integral (SOI) method to address the issue and provide a more quantitatively informative and powerful solution. The basic strategy involves decomposing a highly coupled system consisting of *N* element cavities into as many as possible pairs of two element cavities. For a pair, each eigenmode mostly confines the electric fields within its cavity region; however, some portions of the fields extend outside the cavity and are evanescently distributed over the space near the other cavity. Notably, the SOI method focuses on integrating this spatial field overlap. This is because the intercavity coupling is a direct manifestation of the field interaction occurring in the space where the two eigenmodes coexist.

**Figure 2:**
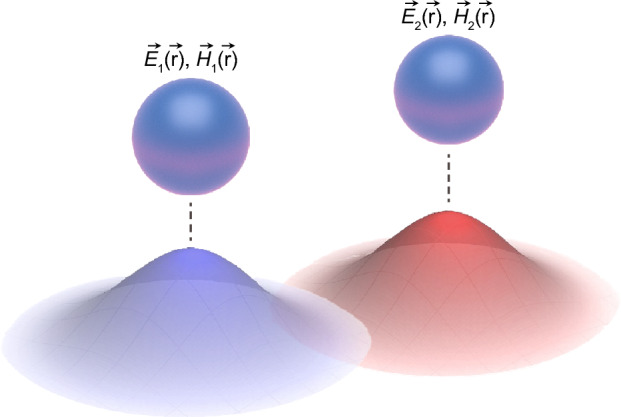
Schematic of coupled element cavities. Electric and magnetic fields of cavities 1 and 2 are ${\overrightarrow{E}}_{i={1,2}}(\overrightarrow{r})$ and ${\overrightarrow{H}}_{i={1,2}}(\overrightarrow{r})$. Blue- and red-colored three-dimensional (3D) Gaussian surfaces schematically represent the spatial distributions of field intensities of the resonant modes excited in cavities 1 and 2, respectively.

To clarify the concept, we consider two adjacent element cavities, 1 and 2, as shown in Fig. 2. The CMT then dictates that the total electric and magnetic fields in this doubly coupled cavity can be expressed as a linear combination of those of the individual cavities as follows:

3 $$ \begin{aligned} \vec{E}(\vec{r},t) & = a_{1} (t)\vec{E}_{1} (\vec{r}) + a_{2} (t)\vec{E}_{2} (\vec{r}) \\ \vec{H}(\vec{r},t) & = b_{1} (t)\vec{H}_{1} (\vec{r}) + b_{2} (t)\vec{H}_{2} (\vec{r}) \\ \end{aligned} $$

Here, $\vec{E}$($\vec{r},t$) ($\vec{H}$($\vec{r},t$)) represents the total electric (magnetic) field of the system, and $\vec{E}_{n = 1,2} (\vec{r})$ ($\vec{H}_{n = 1,2} (\vec{r})$) exhibits a complex electric (magnetic) field of the original eigenmode supported by the element cavity. In addition, $a_{n = 1,2} (t)$ ($b_{n = 1,2} (t)$) is the time-dependent amplitude of the corresponding electric (magnetic) field that varies harmonically with time: $a_{n} (t) = A_{n} e^{{ - i(\omega_{i} t + \varphi_{i} )}}$, and $b_{n} (t) = B_{n} e^{{ - i(\omega_{i} t + \varphi_{i} )}}$.

For two weakly coupled identical dielectric cavities with the same eigenfrequency $$f_{0} $$ and spatial field distribution, we can obtain a set of coupled differential equations for $$a_{n = 1,2} (t)$$ and $$b_{n = 1,2} (t)$$ by exploring Maxwell’s equations and applying orthogonality conditions between fields as follows^25,26^:

4 $$ \begin{aligned} \frac{{da_{1} }}{dt} & = - \frac{2\pi i}{{g^{2} }}\left[ {f_{0} cgb_{1} + f_{0} \left( {c^{\prime}g - cg^{\prime}} \right)b_{2} } \right] \\ \frac{{da_{2} }}{dt} & = - \frac{2\pi i}{{g^{2} }}\left[ {f_{0} \left( {c^{\prime}g - cg^{\prime}} \right)b_{1} + f_{0} cgb_{2} } \right] \\ \end{aligned} $$

5 $$ \frac{{db_{1} }}{dt} = - 2\pi if_{0} a_{1} ,\quad \frac{{db_{2} }}{dt} = - 2\pi if_{0} a_{2} $$

where

6 $$ \begin{aligned} c & = \smallint \varepsilon_{s} \left| {\vec{E}_{1} } \right|^{2} dV, c^{\prime} = \smallint \varepsilon_{s} \vec{E}_{1}^{*} \cdot \vec{E}_{2} dV \\ g & = \smallint \varepsilon_{c} \left| {\vec{E}_{1} } \right|^{2} dV, g^{\prime} = \smallint \varepsilon_{c} \vec{E}_{1}^{*} \cdot \vec{E}_{2} dV \\ \end{aligned} $$

Here, $\varepsilon_{s}$ is the permittivity of cavity 1, and $\varepsilon_{c}$ is the permittivity of the coupled cavity system and the integrations are applied to all space. By combining Eqs. (4) and (5), we can readily eliminate $b_{n = 1,2} (t)$ and rewrite the coupled equations using the following second-order differential equations for $a_{n = 1,2} (t)$:

7 $$ \begin{aligned} & \frac{1}{{4\pi^{2} }}\frac{{d^{2} a_{1} }}{{dt^{2} }} + \frac{c}{g}f_{0}^{2} a_{1} + \frac{c}{g}f_{0}^{2} \left( {\frac{c^{\prime}}{c} - \frac{g^{\prime}}{g}} \right)a_{2} = 0 \\ & \frac{1}{{4\pi^{2} }}\frac{{d^{2} a_{2} }}{{dt^{2} }} + \frac{c}{g}f_{0}^{2} a_{2} + \frac{c}{g}f_{0}^{2} \left( {\frac{c^{\prime}}{c} - \frac{g^{\prime}}{g}} \right)a_{1} = 0 \\ \end{aligned} $$

Considering the fact that the field amplitudes harmonically change with time, the coupled differential equations provide the two eigen-solutions for $\omega$: the higher and lower values are $\omega_{ + }$ and $\omega_{ - }$, respectively. Consequently, the coupling coefficient $\kappa_{c}$ between the two cavities can be expressed in the form of field overlap integrals^27^; that is,

8 $$ \kappa_{c} = \frac{{\omega_{ + }^{2} - \omega_{ - }^{2} }}{{\omega_{ + }^{2} + \omega_{ - }^{2} }} = \frac{c^{\prime}}{c} - \frac{g^{\prime}}{g} $$

In addition, if the interaction between the two cavities is sufficiently weak, the expression for $\kappa_{c}$ in Eq. (8) can be further approximated as a simpler form:

9 $$ \kappa_{c} \cong \frac{{\omega_{ + } - \omega_{ - } }}{{\omega_{0} }} $$

Finally, the expression for $\kappa_{c}$ can be directly converted into the coupling constant $J$; that is,

10 $$ J = \frac{{f_{ + } - f_{ - } }}{2} = \frac{{f_{0} }}{2}\kappa_{c} = \frac{{f_{0} }}{2}\left( {\frac{c^{\prime}}{c} - \frac{g^{\prime}}{g}} \right) $$

As a result, we have successfully obtained the overlap integral expression for the coupling constant in a doubly coupled cavity system. Significantly, the result can be used to provide all the necessary components in Eq. (2) if we properly decompose the system into a set of doubly coupled systems and independently calculate the corresponding $J_{n,m}$. Consequently, it allows us to achieve the complete eigen-solutions for the systems with *n* ≥ 3. In the next section, we consider doubly coupled lossless passive nanocavities to apply the SOI method and examine its feasibility by directly comparing the results with those obtained independently from the frequency method.

### Intercavity interactions in doubly coupled PhC nanocavities

**Figure 3:**
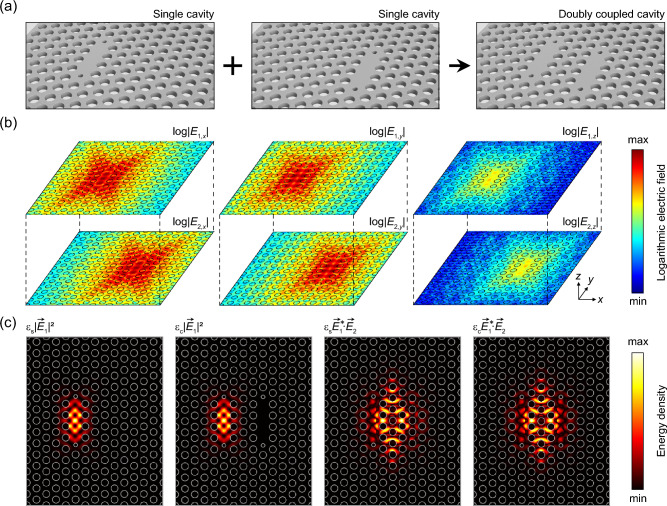
Overlap integral method in coupled nanocavities. (**a**) Schematics showing two identical L3 PhC nanocavities (left, middle) forming a doubly coupled nanocavity (right). (**b**) Logarithmic plots of all three electric field components of a resonant mode in each element cavity: *x*-, *y*-, and *z*-components (from left to right). (**c**) Plots of the integrands in overlap integral expressions: $c$, $c{\prime}$, $g$, and $g{\prime}$ (from left to right).

Among the various types of optical resonant cavities, we preferentially employed doubly coupled photonic crystal (DC-PhC) nanocavities as our test-bed system (Fig. 3)^17^. Because a nanocavity with a submicron footprint typically supports a few eigenmodes with a large free spectral range (FSR), one can readily identify the eigenmodes and easily trace their spectral behaviors. The nanocavity used in this study is called the modified L3 PhC nanocavity, which was constructed as a two-dimensional slab structure with a thickness of 250 nm. A linear defect was introduced by missing three consecutive airholes and reducing two nearest-neighbor airholes in a triangular lattice PhC structure. The lattice constant was 420 nm, and the regular and reduced airhole sizes were 265 nm and 140 nm, respectively. The refractive index of the slab was set to 3.3. The two reduced air holes were outwardly shifted by 63 nm from their original lattice positions, effectively enlarging the cavity volume^17^.

As shown in Fig. 3a, a horizontally coupled PhC nanocavity can be considered a combination of two identical element nanocavities separated by three rows of airholes. To describe the process of the overlap integral method, we first excited a eigenmode in each element nanocavity and investigated the electric field distributions. For the numerical simulation, we used the finite element method (FEM, COMSOL Multiphysics 5.6, wave optics module, COMSOL Inc.), which solves the full three-dimensional (3D) Helmholtz equation under appropriate structural parameters and boundary conditions. The logarithmic plots of *x*-, *y*-, and *z*-components of the electric fields clearly show the strong field confinement within the nanocavity and the evanescently decaying fields distributed over the space (Fig. 3b). This result indicates that the evanescently extended fields from each cavity can sufficiently overlap when the two cavities are closely coupled, thus allowing rich interaction. These interactions can be parameterized by calculating the extent of overlap in both amplitude and space. Furthermore, we show the spatial field distributions of integrands in $c$, $c^{\prime}$, $g$, and $g^{\prime}$ of Eq. (8) to clarify the process (Fig. 3c).

**Figure 4:**
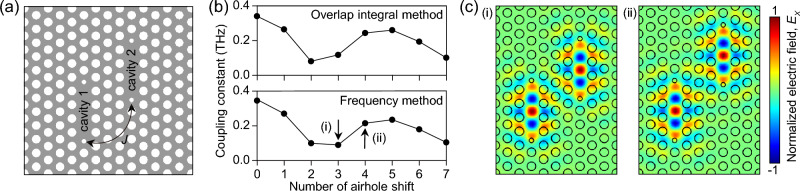
(**a**) DC-PhC nanocavity with the vertical shift of one of the element cavities. (**b**) Calculated coupling constants as a function of the vertical airhole shift using the overlap integral method (top) and the frequency method (bottom). (**c**) Electric field profiles obtained from the three (i) and four airhole shifts (ii) displaying different field interactions.

To verify the feasibility of the SOI method, we systematically changed the coupling geometry of the cavity (Fig. 4) and independently calculated the coupling constant $J$ using the SOI and the frequency methods, respectively. In Fig. 4a, one of the element cavities (cavity-2) gradually shifts upward, whereas the other remains unchanged. The $J$ calculated by the SOIs are plotted as a function of airhole shift (top, Fig. 4b).

As expected, the interaction started decreasing initially but bounced back and reached a local maximum when five airholes were shifted. This is because the symmetry of the original eigenmode and the correspondingly distributed fields largely overlap when the lower boundary of cavity-2 becomes closer to the upper boundary of cavity-1 ((i) and (ii), Fig. 4c). These observations, including the overall features and detailed values of the plot, were reproduced almost identically using the frequency method (bottom, Fig. 4b). Therefore, we conclude that the SOI approach can be another powerful numerical method for describing intercavity interactions and can be more widely applied to coupled systems with multiple element cavities.

### SOI analysis on triply coupled PhC nanocavities

**Figure 5:**
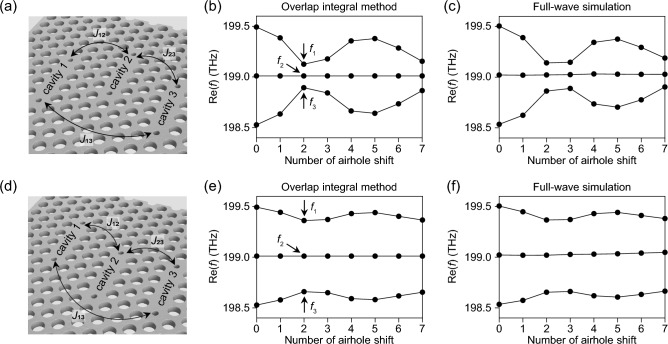
Overlap integral analysis on TC-PhC nanocavities. (**a**) TC-PhC nanocavities with a central element cavity being shifted upward. (**b**,**c**) Calculated system eigenfrequencies as a function of the number of airhole shift using the overlap integral method (**b**) and the full-wave simulation (**c**). (**d**) TC-PhC nanocavities with left element cavity being shifted upward. (**e**,**f**) The same overlap integral and full-wave simulation analysis for the structures of (**d**).

Based on the results shown in Fig. 4, we further increased the level of complexity in intercavity coupling by introducing another element cavity (cavity-3) in the horizontal direction (Fig. 5). This three-coupled (or triply coupled) PhC (TC-PhC) nanocavity system allows for two different methods of systematic variation in the coupling geometry. One is to shift the central element cavity (cavity-2) upward (Fig. 5a), and the other is to shift either the left (cavity-1) or right element cavity (cavity-3) upward (Fig. 5d).

Figure 5b,e show the normal mode eigenfrequencies calculated as functions of the number of airhole shifts using the SOI method. As expected, the shift in the central element cavity had more dramatic effects on the changes in the eigenfrequencies, exhibiting more distinct and structured features in the plot. This is because the shift in Fig. 5a equally affects the field interactions between element cavities 1 and 2 ($J_{12}$) and that of 2 and 3 ($J_{23}$), whereas the shift in Fig. 5d is expected to exclusively influence $J_{12}$. This difference becomes more noticeable when it reaches a critical point, at which the amount of change in the field interaction is greater than that at other shift positions. For example, when the shift was made by two airholes, the field interaction between adjacent cavities was significantly reduced owing to the symmetry of the original eigenmode. Subsequently, it applied to both $J_{12}$ and $J_{23}$, resulting in all normal mode eigenfrequencies being closest to the original eigenfrequency ($f_{0}$) (Fig. 5b). However, this effect decreased by half in the case of Fig. 5e because $J_{23}$ remained almost unchanged.

**Figure 6:**
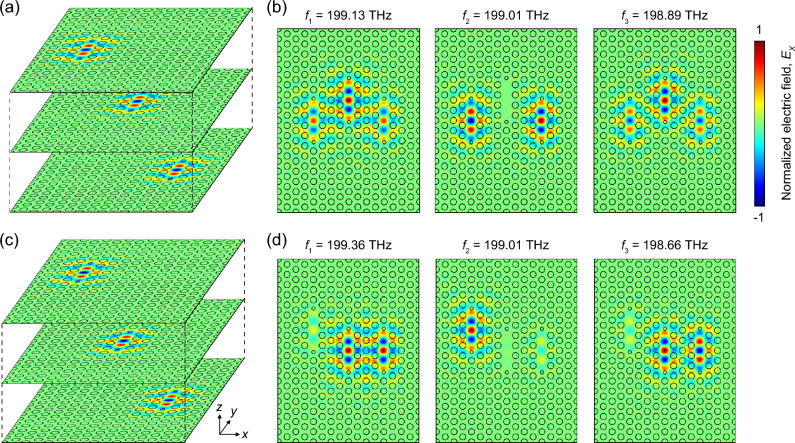
Field profile generation using overlap integral method. (**a**,**c**) Consolidation process to obtain *E*_*x*_-field profiles in the TC-PhC nanocavities of Fig. 5a,d with two airhole shift using overlap integral method. (**b**) Three system eigenmodes at 199.13 THz (${f}_{1}$), 199.01 THz (${f}_{2}$), and 198.89 THz (${f}_{3}$) for the nanocavity of Fig. 5a (arrows in Fig. 5b). (**d**) Three system eigenmodes at 199.36 THz (${f}_{1}$), 199.01 THz (${f}_{2}$), and 198.66 THz (${f}_{3}$) for the nanocavity of Fig. 5d (arrows in Fig. 5d).

Unambiguously, the electric field profiles strongly support our analysis (Fig. 6). The field profiles of the coupled nanocavities were generated by consolidating the original eigenmode field profiles at different element nanocavities weighted by the system’s eigenfrequencies (left, Fig. 6a,c). Figure 6b,d show the generated electric fields of the three normal mode at different eigenfrequencies for the TC-PhC nanocavities shown in Fig. 5a,d, respectively. Furthermore, all these features in the normal mode eigenfrequencies and field profiles were reproduced by full-wave simulations, reconfirming the feasibility and accuracy of the SOI method (Fig. S1).

### SOI analysis on non-Hermitian coupled nanocavity systems

We can further expand the application of the SOI analysis to a more complex non-Hermitian coupled nanocavity system. In this case, the gains ($\gamma_{n}$) and losses ($\kappa_{n}$) of the individual element cavities are no longer negligible but are restored to different values, leading to a non-symmetrical gain/loss profile within the coupled system. To take full advantage of the results and in-depth understandings in Figs. 5 and 6, we explore the TC-PhC nanocavity structure again; however, different levels of optical gains are introduced on the individual element nanocavities ($\gamma_{1} \ne \gamma_{2} \ne \gamma_{3} \ne 0$, Fig. 6). The characteristic matrix equation describing the system is then written as

11 $$ \frac{i}{2\pi }\frac{d}{dt}\left[ {\begin{array}{*{20}c} {a_{1} } \\ {a_{2} } \\ {a_{3} } \\ \end{array} } \right] = \left[ {\begin{array}{*{20}c} {f_{0} + i(\gamma_{1} - \kappa )} & {J_{12} } & 0 \\ {J_{12} } & {f_{0} + i(\gamma_{2} - \kappa )} & {J_{23} } \\ 0 & {J_{23} } & {f_{0} + i(\gamma_{3} - \kappa )} \\ \end{array} } \right]\left[ {\begin{array}{*{20}c} {a_{1} } \\ {a_{2} } \\ {a_{3} } \\ \end{array} } \right] $$

For simplicity, we set the intrinsic loss $\kappa$ identical to all three element nanocavities (i.e., $\kappa_{1} = \kappa_{2} = \kappa_{3} = \kappa$). In addition, the time-reversal condition and weak-coupling assumption were applied to the equation.

**Figure 7:**
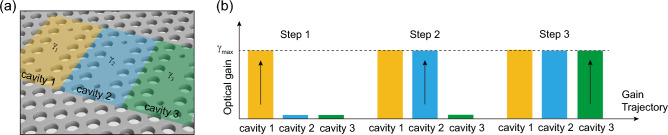
Non-Hermitian TC-PhC nanocavities with asymmetric optical gains. (**a**) Schematic showing three different optical gains assigned to element cavities. The yellow, blue, and green shaded areas indicate regions where different optical gains with a volume of 1.46 × 2.52 × 0.25 µm^3^ are introduced. (**b**) Sequential steps of introducing optical gains to element cavities.

Figure 7a shows the non-Hermitian TC-PhC nanocavity; the optical gains of $\gamma_{1}$, $\gamma_{2}$, and $\gamma_{3}$ are introduced to element nanocavities of 1 (yellow), 2 (blue) and 3 (green), respectively. Next, we sequentially varied the individual optical gains $\gamma_{n}$ (Fig. 7b); Step 1: for $\gamma_{2} = \gamma_{3} = 0$, the $\gamma_{1}$ increases gradually from 0 to $\gamma_{{{\text{max}}}}$, Step 2: for $\gamma_{1} = \gamma_{{{\text{max}}}}$ and $\gamma_{3} = 0$, the $\gamma_{2}$ increases gradually from 0 to $\gamma_{{{\text{max}}}}$, and Step 3: increasing $\gamma_{3}$ from 0 to $\gamma_{{{\text{max}}}}$, while $\gamma_{1}$ and $\gamma_{2}$ is fixed at $\gamma_{{{\text{max}}}}$. In such non-Hermitian coupled systems, it is known that a critical level of non-uniformity in optical gain/loss often invites unexpected transitions in phase, resulting in unprecedented optical phenomena, such as modal coalescence and the sudden emergence of bifurcations. Significantly, these unusual events are reflected on the system’s eigenfrequencies; therefore, it is significantly importance to investigate the spectral trajectories of the system.

**Figure 8:**
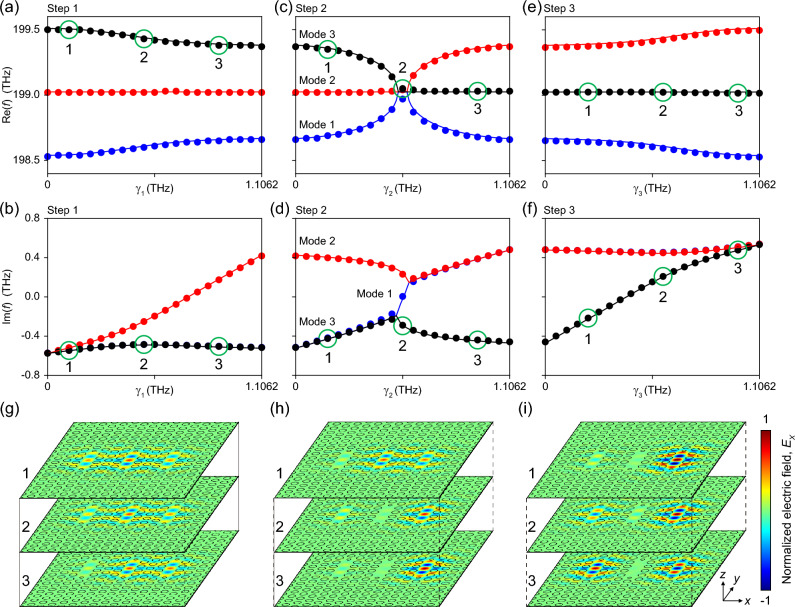
Eigenfrequencies and field profiles of non-Hermitian TC-PhC nanocavities. (**a**–**f**) The calculated real (upper panels) and imaginary parts (lower panels) of eigenfrequencies obtained by overlap integral (lines) and frequency methods (circles). (**g**–**i**) Logarithmic plots of electric field profiles of a selected mode following the sequential steps 1 (**a**,**b**), 2 (**c**,**d**), and 3 (**e**,**f**), respectively. In each step, field profiles of three different eigenfrequencies indicated 1, 2, and 3 (green circles) were plotted to show how the mode develops with the gain.

In Fig. 8, we have plotted the real (Re($f$)) and imaginary parts (Im($f$)) of all normal mode eigenfrequencies as well as the field profiles of the selected normal modes as the gain sequentially varies. There are several interesting non-Hermitian features. First, in the entire process of Step 1, the system supported three normal modes (Modes 1 (blue), 2 (red), and 3 (black)), which were spectrally well separated (Fig. 8a). This indicates that the system remained in an unbroken parity-time (PT) symmetric phase. Second, as $\gamma_{1}$ increased, Mode 1 changed from the decaying type to the oscillating type and further changed to the amplifying type mode (red lines and circles, Fig. 8a,b) without a significant shift in its spectral position. The other two eigenmodes exhibited almost identical Im($f$) values (blue and black lines and circles). Third, as expected, Step 3 was a simple reverse process of Step 1; therefore, similar features but in the reverse direction were observed (Fig. 8e,f). Fourth, in Step 2, the phase transition, one of the most unique non-Hermitian properties, occurred at exceptional points (EPs), resulting in the normal mode coalescence and the emergence of modal trifurcation (Fig. 8c,d). Furthermore, a gradual evolution from the decaying- to the amplifying-type mode was observed in Mode 1 (Fig. 8d). Interestingly, as $\gamma_{2}$ increased, the Re($f$) of Mode 1 exhibited an overall symmetric feature with a local maximum at the EP (Fig. 8c). In addition, no mode-type transition has occurred in the other two modes; however, their Re($f$) exhibited mirror-like behaviors with respect to the EP: (1) as $\gamma_{2}$ approached to EP, Re($f$) of Mode 2 remained unchanged, whereas those of Mode 3 rapidly fell onto the EP and (2) as $\gamma_{2}$ further increased from EP to $\gamma_{{{\text{max}}}}$, these observation occurred vice versa. Significantly, these features are unique non-Hermitian behaviors that cannot be observed even in doubly coupled non-Hermitian systems. In addition, although some of the features originate from the choice of coupled cavity structure, most features, including phase and mode-type transitions, are well-known characteristics observed in non-Hermitian coupled systems. We believe that the proposed multiply-coupled nanocavity systems are not only intriguing in a stand-alone form but also useful for designing unique optical applications when integrated (or combined) with other optical components (techniques). For example, a programmable adaptive pumping enables to deliver two-dimensional (2D) phase/intensity information of pumping beam to coupled nanocavities and realize the site-controlled operation of lasing between cavities. In addition, an aligned micro-transfer can further realize programmable light coupling to optical waveguides, which directly allows for encode the 2D phase/intensity maps of incident beam to transmitted optical signals at the ends of waveguides.

## Discussion

We investigated the intercavity interactions in various coupled PhC nanocavities with different coupling schemes. We showed that the coupling constant can be determined using the SOI method, which can facilitate normal mode analysis without increasing the levels of complexity. It was demonstrated that the original eigenmode frequency and field profile of a single cavity can be used as building blocks to determine the normal modes of coupled cavities. Furthermore, we successfully demonstrated that non-Hermitian TC-PhC nanocavities with different coupling geometries could be analyzed using the SOI method. We believe that the SOI method can help to understand important characteristics of various non-Hermitian optical systems.

### Supplementary Information

Supplementary Figure S1.

## Data Availability

The datasets used and/or analyzed during the current study are available from the corresponding author on reasonable request.
