# Supplementary material for: Normal mode analysis in multi-coupled non-Hermitian optical nanocavities
Source: Sci Rep. 2023 Oct 16;13:17510. doi: 10.1038/s41598-023-44809-w (PMC10579268; doi:10.1038/s41598-023-44809-w)
Supplement: Supplementary file 1 — Supplementary Figure S1. [file 41598_2023_44809_MOESM1_ESM.docx]

Normal mode analysis in multi-coupled non-Hermitian optical nanocavities

Kyong-Tae Park^1,†^, Kyoung-Ho Kim^2,†^, Byung-Ju Min^1^ and You-Shin No^1,*^

^1^Department of Physics, Konkuk University, Seoul 05029, Republic of Korea

^2^Department of Physics, Chungbuk National University, Cheongju 28644, Republic of Korea

**Corresponding Author**

^*^E-mail: ysno@konkuk.ac.kr

Supplementary Information Figures S1


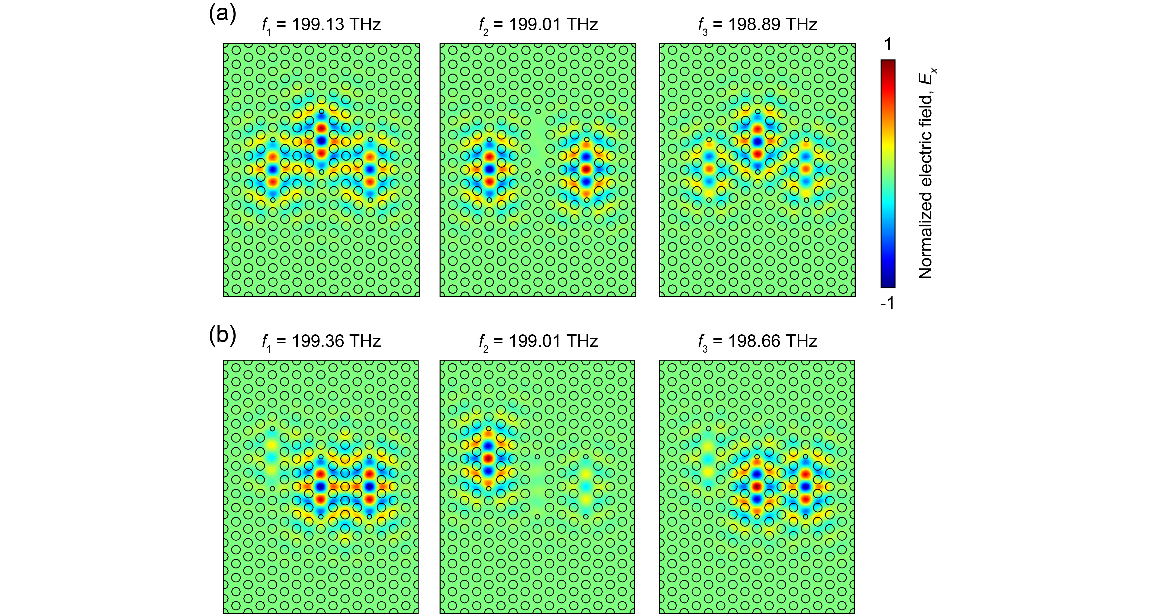


Fig. S1. Field profiles of TC-PhC nanocavities obtained from the full-wave simulations.
